# Differences in bleeding behavior after endoscopic band ligation: a retrospective analysis

**DOI:** 10.1186/1471-230X-10-5

**Published:** 2010-01-15

**Authors:** Florian Petrasch, Johannes Grothaus, Joachim Mössner, Ingolf Schiefke, Albrecht Hoffmeister

**Affiliations:** 1 Department of Internal Medicine II, University of Leipzig, 04103 Leipzig, Germany

## Abstract

**Background:**

Endoscopic band ligation (EBL) is generally accepted as the treatment of choice for bleeding from esophageal varices. It is also used for secondary prophylaxis of esophageal variceal hemorrhage. However, there is no data or guidelines concerning endoscopic control of ligation ulcers. We conducted a retrospective study of EBL procedures analyzing bleeding complications after EBL.

**Methods:**

We retrospectively analyzed data from patients who underwent EBL. We analyzed several data points, including indication for the procedure, bleeding events and the time interval between EBL and bleeding.

**Results:**

255 patients and 387 ligation sessions were included in the analysis. We observed an overall bleeding rate after EBL of 7.8%. Bleeding events after elective treatment (3.9%) were significantly lower than those after treatment for acute variceal hemorrhage (12.1%). The number of bleeding events from ligation ulcers and variceal rebleeding was 14 and 15, respectively. The bleeding rate from the ligation site in the group who underwent emergency ligation was 7.1% and 0.5% in the group who underwent elective ligation. Incidence of variceal rebleeding did not vary significantly. Seventy-five percent of all bleeding episodes after elective treatment occurred within four days after EBL. 20/22 of bleeding events after emergency ligation occured within 11 days after treatment. Elective EBL has a lower risk of bleeding from treatment-induced ulceration than emergency ligation.

**Conclusions:**

Patients who underwent EBL for treatment of acute variceal bleeding should be kept under medical surveillance for 11 days. After elective EBL, it may be reasonable to restrict the period of surveillance to four days or even perform the procedure in an out-patient setting.

## Background

Portal hypertension is a hemostatic disorder that is often caused by liver cirrhosis. It is defined as an increase in blood pressure in the portal venous system. Portal hypertension can be the origin of severe complications in cirrhosis. Gastroesophageal varices are a potentially life-threatening complication of portal hypertension. Even though the outcome of bleeding from gastroesophageal varices has improved, it is still associated with a high mortality rate. The mortality rate of acute variceal bleeding has decreased from 42% in 1981 to about 15% to 20% at present [1-4].

Risk factors for bleeding from esophageal varices have been identified in several randomized controlled trials (RCT). Poor Child-Pugh score [3,5-7], bacterial infection [6-8], elevated aspartate amino transferase levels [3], and a hepatic venous pressure gradient greater than 20 mmHg, measured shortly after admission [5,6,8-10], are associated with a higher bleeding rate.

Management of acute variceal bleeding consists of pharmacologic and endoscopic treatment. Endoscopic techniques currently used to stop variceal bleeding are endoscopic sclerotherapy, variceal obturation with glue and endoscopic band ligation (EBL). EBL is the endoscopic treatment of choice because it is an effective treatment for bleeding from esophageal varices. It is generally accepted and recommended for the treatment of acute bleeding events. EBL is also used for the secondary prophylaxis of esophageal variceal hemorrhage [11-15].

Non-selective beta-blockers are the treatment of choice to prevent initial bleeding episodes. However, beta-blockers do not prevent formation of esophageal varices. For primary prophylaxis of esophageal bleeding, EBL should be restricted to patients with contraindications or intolerance to beta-blockers [14,16-19].

EBL sessions are usually repeated at 1-2 week intervals until complete obliteration of all esophageal varices has been achieved [15]. Complete obliteration is achieved in about 90% of patients after 2-4 sessions. Variceal recurrence occurs frequently, with 20%-75% of patients requiring repeated EBL sessions.

There is numerous data, including randomized controlled trials and meta-analyses, concerning the safety of EBL and its clinical effectiveness in controlling variceal bleeding. However, there are only few studies that have evaluated the risk of bleeding from ligation-induced ulcers. The results of these few studies are contradictory [19-21]. Stiegmann et al. [21] reported an overall complication rate of only 2%. Schepke et al. [19] completed a multicenter trial comparing EBL with beta-blocker therapy for primary prophylaxis of variceal bleeding. This study found the incidence of bleeding from ligation ulcers after EBL to be 6.7%. Another retrospective data analysis of EBL described hemorrhage from ligation ulcers as 5.7%, irrespective of the indication [20].

Meetings were held to discuss clinical trials and propose guidelines for the management of patients with esophageal varices. During the proceedings at the Baveno IV workshop in April 2005, attendees came to an agreement about the management of endoscopic treatment of esophageal varices [14]. However, as far as we know, there is no data or guidelines concerning endoscopic control of ligation ulcers. Furthermore, there is no data or guidelines on whether EBL should be restricted to in-patients. Most gastroenterologists, at least in Germany, do not perform out-patient EBL. Patients are kept under surveillance until all ligation bands are dropped off. This monitoring is based on the theory that the process of dropping off ligation bands is associated with an elevated risk of recurrent bleeding.

EBL is routinely used in nearly all gastrointestinal endoscopy units. Tremendous efforts are invested in the therapy of esophageal varices. Despite this, there is no evidence how to monitor patients after EBL. Gastroenterologists, like all other physicians, are increasingly under economic pressure. German health insurance funds and hospital administration executives try continuously to further minimize the duration of hospitalizations. We therefore conducted a retrospective study of endoscopic band ligation procedures with regard to the primary outcome of bleeding complications after EBL. Furthermore we wanted to evaluate whether ligation ulcers after EBL are a locus of high risk for procedure associated bleeding events.

## Methods

### Patient characteristics

**Figure 1:**
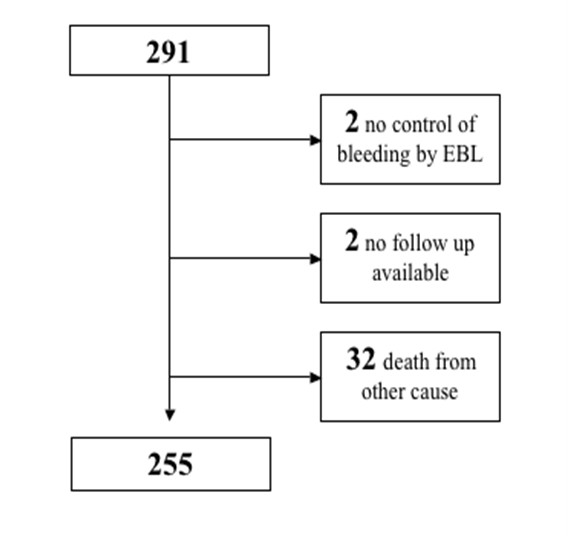
**Patients included in the data analysis**. From the 291 patients who underwent EBL during the observation period, 255 could be enrolled in the study.

Our retrospective analysis included all patients who underwent endoscopic band ligation of esophageal varices between July 1, 2000 and January 31, 2007 at our institution. A total of 291 patients with esophageal varices have been treated. In these patients, 430 ligature procedures have been performed. For patient characteristics, see figure 1.

Endoscopic and hospital records were reviewed to obtain demographic, clinical, endoscopic and follow-up data. Data for the analysis included indication for endoscopic procedure, bleeding events, time interval between EBL and bleeding, and time interval between endoscopic procedure and dropping off of ligation bands.

Acute bleeding events from varices were defined as active variceal bleeding (visible oozing or spurting of blood from a varix) or the presence of stigmata of recent bleeding in the presence of varices of grade two or higher. Stigmata of recent hemorrhage was defined as the presence of an adherent clot, white protrusion, varices and blood in the stomach, or large varices in the setting of a patient with hematemesis and no other cause of upper gastrointestinal bleeding.

All patients were hospitalized until drop-off of all ligation bands, proven by endoscopy. Patients were divided into two subgroups according to their indication for endoscopic treatment. The first subgroup consisted of patients who underwent banding ligation because of an acute event of esophageal variceal hemorrhage. The second subgroup included patients who underwent elective primary or secondary treatment for prevention of bleeding. Bleeding was defined as the following: (1) a bleeding event after initial control of variceal bleeding, (2) a bleeding event after successful EBL of non-bleeding varices before all ligation bands dropped off. Any patient with clinical symptoms of bleeding (i.e., new hematemesis, melena, tachycardia, hypotension, or insufficient increase in hemoglobin after erythrocyte substitution) was admitted for a new endoscopic procedure. The cause of every incident of gastrointestinal bleeding was evaluated by endoscopy. The diagnosis of bleeding from the varices or from ligation ulcers was accepted if active bleeding was identified, a clot was seen adherent to a ligation site, or blood was detected in the upper GI-tract and no other cause of bleeding from the gastrointestinal tract was evident.

### Endoscopic variceal ligation and further interventions

Upper gastrointestinal endoscopy and esophageal variceal band ligation was performed in all patients using a high-resolution video endoscope (Olympus, Hamburg, Germany) loaded with a Multiband ligator (Wilson-Cook Medical). Only experienced endoscopists performed the ligations. All endoscopists had received training of at least one year. After training, these endoscopists were only allowed to independently treat varices after they had performed a minimum of ten EBL procedures under surveillance of more experienced endoscopists. Ligation was applied beginning one centimeter above the gastroesophageal junction. Each varix was ligated until the bleeding stopped, the varices were eradicated, or the varices were reduced to grade 1. Further sessions of treatment followed in cases of recurring or persisting varices.

### Data analysis

Statistical analysis was performed using the SPSS software package (SPSS Inc., Chicago IL). Basic descriptive statistics (means, standard deviations, ranges, medians, and percentages) were used to characterize the patient's cohort. Variceal bleeding incidence after endoscopic ligation was evaluated with contingency tables, including χ^2 ^statistics, Fisher's exact test and Mann-Whitney-U-Test. The Kaplan-Meier method was used to estimate the rates of survival without bleeding. The log-rank test was used to test the significance between the two arms. A two tailed p-value less than 0.05 was considered significant in the assessment of modus of ligation, bleeding from ligation-sites, and associations between hemorrhage and other recorded factors. Kaplan-Meier estimations were completed to analyze the time until new bleeding episode. This analysis was done separately for the groups with recurrent variceal hemorrhage and those with bleeding from their ligation site.

All data were managed anonymised. The local ethics committee confirmed that no ethical approval was needed for this study.

## Results

A total of 255 patients with an average age of 57.3 ± 12.6 years were included in the analysis. We performed 218 sessions of EBL to control acute hemorrhage and 212 for primary (31 sessions) or secondary (181 sessions) prevention. Two patients were excluded from analysis because their follow-up data was not available due to referral to other hospitals. Additionally, two other patients were excluded because acute variceal bleeding could not be treated sufficiently by EBL. Thirty-two patients died from procedure-independent causes other than esophageal bleeding (Table 1) before all ligation bands were dropped off. The remaining 255 patients underwent 387 ligation sessions. During the follow-up period, 30 patients of this cohort had a bleeding event related to ligation ulcers or recurrent esophageal varices. Basic characteristics of these patients are shown in Table 2. The number of bleeding events from ligation ulcers and from recurrent varices was 14 and 15, respectively. In one case, it was not possible to retrospectively evaluate the exact source of bleeding from the endoscopic records. All together, patients underwent 387 ligation sessions, with 205 (53%) of these interventions performed electively for primary (31 interventions) or secondary (174 interventions) prevention of bleeding. The remaining 182 (47%) ligation sessions were carried out to control acute bleeding events.

**Table 1:** Death from procedure-independent causes other than esophageal bleeding (n = 32).

Death of septicaemia:	9
Death of haemorrhagic shock by intrabdominal bleeding (bleeding from intestinal tumor, intraabdominal vessels in liver hilus and omentum majus):	3
Death of haemorrhagic shock by gastric or duodenal bleeding:	4
Death of liver failure, hepatorenal syndrom and liver malignoma (HCC: 3, metastases of colon carcinoma: 1, by haemangioendothelioma: 1)	12
Death of acute renal failure due to plasmocytoma	1
Death of pulmonary oedema due to malignant infiltration:	1
Death of cerebral oedema due to brain haemorrhage:	1
Death of ventricular fibrillation:	1

**Table 2:** Clinical characteristics of patients undergoing endoscopic band ligation (n = 255).

	Without bleeding complications (n = 225)	Bleeding from recurrent varices (n = 15)	Bleeding from EBL-ulcers (n = 14)	Bleeding from an unknown location (n = 1)
Age (y):	57.3 ± 12.6	53.5 ± 12.2	51.1 ± 10.4	36 ± 0.0
Sex (M/F):	146/79	9/6	10/4	0/1
Variceal strands:	3.00 ± 1.1	3.5 ± 0.6	3.1 ± 1.1	4.0 ± 0.0
Ligation sessions:	1.4 ± 0.8	2.2 ± 1.1	2.5 ± 1.6	3.0 ± 0.0
Average number of applied rubber bands per patient and session:	6.0 ± 2.5	6.0 ± 3.0	6.4 ± 1.4	6.0 ± 0. 0
Duration of hospital stay after ligation (days)	13.2 ± 9.1	19.0 ± 14.0	21.8 ± 15.4	18 ± 0.0

The analysis of bleeding events after esophageal ligation was based on the location of bleeding, the indication for EBL (Table 3.) and the number of applied ligation bands per session (Tables 4, 5). The mean time interval between EBL and first endoscopy to control dropping off of rubber bands was 7.4 (± 3.0) days for patients who underwent elective EBL and 6.6 (± 3.5) days for patients after emergency procedure.

**Table 3:** Mode of ligation and source of bleeding in performed ligation sessions.

	Elective ligation (n = 205)	Ligation for acute bleeding control (n = 182)	p
Bleedings	8 (3.9%)	22 (12.1%)	0.004
Bleeding at ligation site	1 (0.5%)	13 (7.1%)	< 0.001
Bleeding at recurrent varices	7 (3.4%)	8 (4.4%)	NS
Esophageal bleeding of unknown location	0	1 (0.6%)	NS

**Table 4:** Time intervals and applied ligation bands.

	Elective ligation	Ligations for acute bleeding control
Duration of hospital stay after ligation (days)	12,4 ± 8,4	15.1 ± 10.9
Time interval between ligation and next endoscopy (days)	7.4 ± 3.0	6.6 ± 3.5
Time interval to detected band drop-off (days)	9.3 ± 4.8	9.2 ± 4.8
Avarage number of applied rubber bands per patient and sessions	6.1 ± 2.7	6.1 ± 2.7

**Table 5:** Number of applied ligation bands per session.

	Average of applied ligation bands	p
Sessions without complications (n = 357):	6.0 ± 2.6	
Bleeding complications altogether (n = 30):	6.8 ± 3.1	NS
Bleeding at ligation site (n = 14):	7.9 ± 2.7	0.009
Bleeding at recurrent varices (n = 15):	5.7 ± 3.7	NS
Esophageal bleeding of unknown location:	9 ± 0	NS

We observed a significant difference in bleeding events when EBL for treatment of acute variceal bleeding was compared to electively performed ligations. The incidence of hemorrhage after acute intervention was 12.1% (22/182) compared to 3.9% (8/205) after elective treatment (p = 0.004). The bleeding rate from ligation sites in the group that underwent emergency ligation was 7.1% (13/182). This was more than fourteen times higher than the group that underwent elective ligation treatment (0.5% (1/205); p < 0.001). However, bleeding incidence from recurrent varices did not vary significantly. Bleeding from recurrent varices occurred in seven patients (3.4%) in the elective group and in eight patients (4.4%) in the emergency group.

The average number of ligation bands applied per session is shown in Tables 4 and 5. Significantly more ligation bands were applied in interventions that were followed by hemorrhage from ligation ulcers (p = 0.009). We found no significant difference in the number of bands applied when we compared those with bleeding from recurrent varices and those who had no bleeding at all.

**Figure 2:**
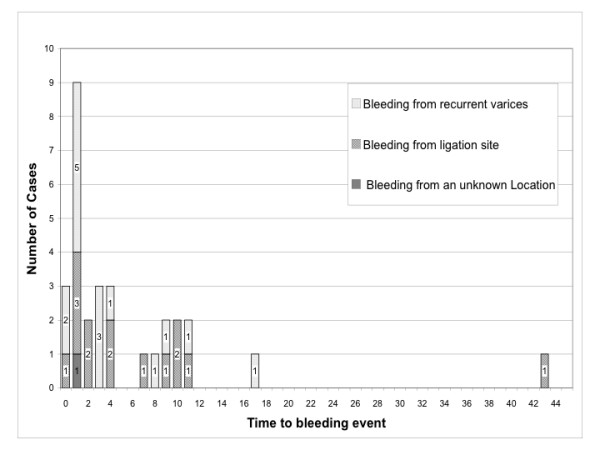
**Distribution of bleeding events**. All documented bleeding events after EBL (n = 30) occurred within 44 days after EBL. 15 bleeding events were observed from recurrent varices and 14 at ligation sites. In one case, the location of bleeding could not be evaluated.

**Figure 3:**
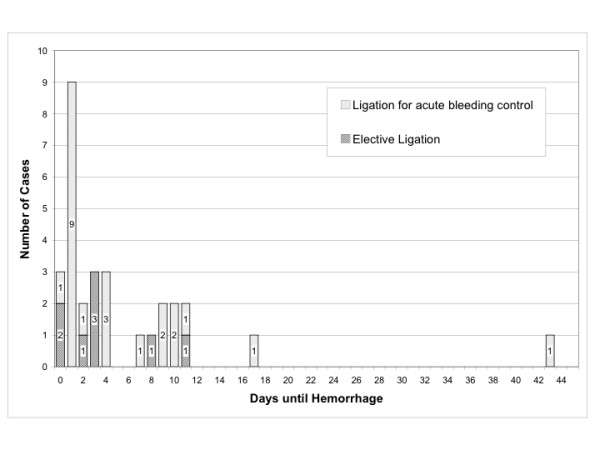
**Ligation mode distribution of hemorrhagic events**. Distribution of bleeding events after EBL for treatment of acute variceal bleeding and after elective EBL.

An analysis of the time until bleeding in all patients showed a predominance of bleeding events during the first four days after endoscopic treatment (figure 2). Sixty-seven percent of bleeding events occurred in this period of time. The majority of these events were hemorrhage from recurrent varices. There were eight events from ligation sites and eleven events from recurrent varices. In addition, there was one hemorrhage without clear determination of localization. Seventy-five percent (6/8) of all bleeding episodes after elective treatment occurred during the first four days after EBL treatment. Most rebleedings after emergency ligation were also observed within the first 4 days, i.e. sixty-four percent (14/22) (figure 3). The latest report of a bleeding after elective ligation treatment was on the 11th day of the follow-up period.

**Figure 4:**
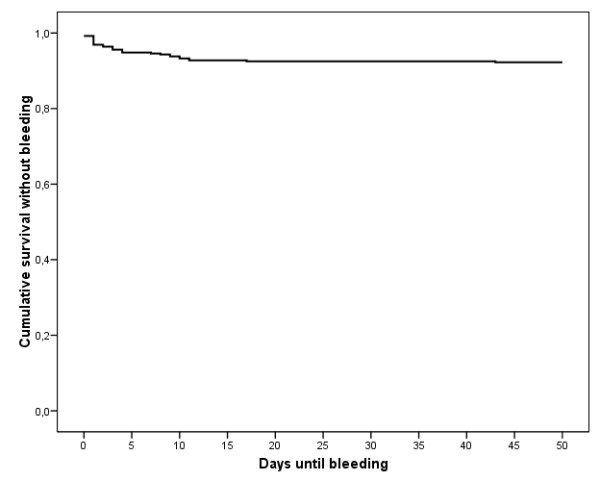
**Kaplan-Meier estimation of cumulative survival without hemorrhage**. The overall risk of bleeding after EBL is reduced from 7.8% to 2.6% after four days, and to 0.5% after 11 days.

**Figure 5:**
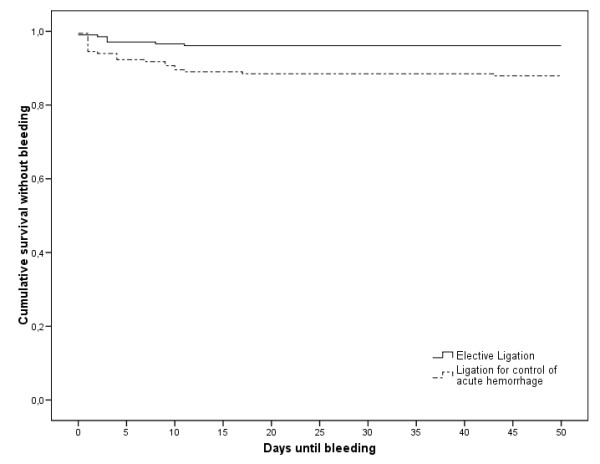
**Kaplan-Meier estimation of cumulative survival without hemorrhage for elective and emergency EBL**. The bleeding risk four days after emergency EBL is significantly higher than after elective treatment, p = 0.04.

Irrespective of the indication for EBL or the localization of bleeding, the cumulative risk of bleeding events was 7.8%. The initial bleeding risk of 2.6% in the first four days after the endoscopic procedure was reduced to 0.5% after 11 days (figure 4). The cumulative rebleeding risk within the first four days after emergency ligation is 0.077. After electively performed treatment, the cumulative bleeding risk is 0.029. A comparison using the Mann-Whitney-Test determined this difference to be significant (p = 0.04) (figure 5).

## Discussion

Endoscopic band ligation (EBL) is the gold standard for the treatment of acute bleeding of esophageal varices. It is also effective in secondary prophylaxes and in primary prophylaxis for patients who are insensitive to beta-blocking agents. EBL is mostly performed in the in-patient setting. Patients are often hospitalized until endoscopy proves all applied ligation bands have dropped off. However, there is no proven, scientific rational for this strategy.

This single center, retrospective analysis studied 255 patients who underwent endoscopic ligation therapy. The aim of this study was to propose an algorithm for the surveillance of patients after EBL. We documented an overall bleeding rate after EBL of 7.8%. Since this rate is consistent with those reported in other studies (9% to 19%) [22-28], it emphasizes that our results are comparable with those of others. These studies were performed for primary prophylaxes [22,23,25,26,28], secondary prophylaxes [24] and control of acute [27] variceal bleeding. The results should be therefore comparable with results obtained in our collective.

One important result of this study was that elective ligation therapy had a significantly lower incidence of procedure related bleeding events than treatment of acute bleeding events. According to our knowledge, there are no other studies relating the bleeding rate after EBL to its indication.

When ligation bands are dropping off, ligation ulcers may form. This process is thought to be associated with an elevated risk of bleeding complications. For this reason, patients are often hospitalized until all applied ligation bands drop off. We found that bleeding from EBL induced ulcers occurred only in 3.6% (14/387) of ligation sessions. In a meta-analysis comparing EBL with sclerotherapy, Laine et al. [29] reported on bleeding from treatment-induced ulcers in seven studies. These studies had patient populations smaller than ours, and ranged from n = 8 to n = 64 for EBL groups. Overall, the documented bleeding rate from treatment-induced ulcers after EBL in these studies was 3.3% (9/274). In later published studies, this value has been reported as 5.4% to 14.2% in patients who underwent the procedure for control of acute variceal hemorrhage [30-32].

These results are consistent with our study. However, to our knowledge, there is no data correlating the indication for EBL to the bleeding rate of ligation ulcers. In our patients, bleeding from ligation ulcers occurred in 7.1% (13/182) of cases after emergency ligation. After electively performed EBL, bleeding at ligation ulcers occurred only in 0.5% (1/205) of cases. Regarding our entire patient cohort, the incidence of bleeding from ligation-induced ulcers was 3.6% (14/387). This was slightly lower than the bleeding rate from recurrent varices, which was 3.9% (15/387). These findings demonstrate that bleeding from ligation ulcers after EBL is not the most important risk factor for bleeding. These data also show that there is a significant difference in bleeding incidence between ligation-induced ulcers after electively performed ligation and after EBL for treatment of acute hemorrhage.

Our results are supported by data concerning bleeding events in recurrent varices. The overall incidence of 3.9% (15/387) in our study is lower than data reported by others (11.9% to 28.6%) [29-32]. However, these studies were designed to compare different endoscopic techniques for the treatment of acute hemorrhage of esophageal varices. Therefore, they did not consider electively performed EBL. Clinical trials have been performed to analyze the prophylactic potential of endoscopic band ligation in elective interventions. In these studies, the incidence of hemorrhage from recurrent varices ranged from 12.5% to 31.7%. Bleeding from ligation-induced ulcers varied from 2.4% to 6.7% [19,26,33]. This data originates from various studies with different indications for EBL. Therefore, the studies are difficult to compare. Studies often do not report the different sources of bleeding in their patients. The need for data concerning EBL in all indications is important for everyday care. Our study is the first analysis of bleeding risk from treatment-induced ulcers or recurrent varices after EBL that focuses on the indication for the interventions.

We were able to show a correlation between the number of applied ligation bands and the bleeding events from ligation ulcers. In most trials, the total number of applied bands per patient seems to have no correlation with bleeding events. Harewood et al. compared the number of bands per session and the bleeding incidence [24]. In this small collection of 40 patients, no significant correlation between the median number of bands used in EBL sessions and bleeding episodes was found. In our study, patients with bleeding events at ligation sites have been treated with significantly more ligation bands. Therefore, one can assume that application of a higher number of ligation bands to treat esophageal varices is a risk factor for bleeding events. Different studies examined the impact of endoscopic therapy of esophageal varices on portal venous pressure [34-36]. The results are not very consistent. However, Ramirez and coworkers showed in a prospective study that there is no better outcome for patients when more than six bands are applied compared to patients treated with a maximum of six ligation bands [37].

We are aware that our study is only a retrospective data analysis and prospective studies should be conducted in order to evaluate policy on EBL procedures. Ligation procedures enrolled in this study could be considered as a further limitation. We reported the analysis of all ligation sessions performed during the study period, because the aim of our study was the evaluation of the procedure associated risk of bleeding after EBL. One could raise concern about this evaluation because there are patients who underwent more then one EBL procedure and were included more than once in data analysis. We therefore recalculated all data after exclusion of all patients who underwent more than one EBL procedures from re-entry into data analysis after first ligation session. The reported results remained significant. (See "Additional file 1", for graphs see "Additional file 2" and "Additional file 3")

## Conclusion

Our study confirms former reports that have demonstrated the safety and effectiveness of endoscopic band ligation for treatment of esophageal varices. The risk of bleeding from treatment-induced ulceration is lower after elective EBL than after emergency intervention. We, therefore, propose that endoscopists may consider elective EBL as an out-patient procedure. In cases when EBL is performed as an in-patient procedure, one may consider restricting the period of surveillance after elective EBL to four days. Elective EBL should be done until all varices are eradicated. An excessive application of ligation bands should be avoided. However, we propose to keep patients who have undergone endoscopic band ligation due to acute esophageal hemorrhage under medical surveillance for at least 8-11 days.

## Abbreviations

EBL: Endoscopic band ligation; RCT: randomized controlled trials

## Competing interests

The authors declare that they have no competing interests.

## Authors' contributions

FP and JG screened clinical records, collected all clinical data and participated in statistical analysis. JM participated in study coordination and helped to draft the manuscript. IS participated in study design and statistical analysis. AH conceived the study, participated in its design and coordination and prepared the manuscript. All authors read and approved the final manuscript.

## Pre-publication history

The pre-publication history for this paper can be accessed here:

http://www.biomedcentral.com/1471-230X/10/5/prepub

## Supplementary Material

Additional file 1 **Data analysis after of the first EBL procedure of all patients**. All patients who underwent more than one EBL procedures were excluded from re-entry into data analysis after first ligation session and data were analyzed regarding only the first EBL procedure of each patient. Click here for file

Additional file 2 **Kaplan-Meier estimation of cumulative survival without hemorrhage regarding only the first EBL procedure per patient**. All patients who underwent more than one EBL procedures were excluded from re-entry into data analysis after first ligation session and Kaplan-Meier estimates were evaluated regarding only the first EBL procedure of each patient. The overall risk of rebleeding after EBL is reduced from 11.8% to 3.9% after four days, and to 0.8% after 11 days. Click here for file

Additional file 3 **Kaplan-Meier estimation of cumulative survival without hemorrhage for elective and emergency EBL regarding only the first EBL procedure per patient**. All patients who underwent more than one EBL procedures were excluded from re-entry into data analysis after first ligation session and Kaplan-Meier estimates were evaluated regarding only the first EBL procedure of each patient. The bleeding risk four days after emergency EBL is significantly higher than after elective treatment, p = 0.042. Click here for file
