# Supplementary material for: Differences in bleeding behavior after endoscopic band ligation: a retrospective analysis
Source: BMC Gastroenterol. 2010 Jan 15;10:5. doi: 10.1186/1471-230X-10-5 (PMC2827370; doi:10.1186/1471-230X-10-5)
Supplement: Additional file 1 — Data analysis after of the first EBL procedure of all patients. All patients who underwent more than one EBL procedures were excluded from re-entry into data analysis after first ligation session and data were analyzed regarding only the first EBL procedure of each patient. [file 1471-230X-10-5-S1.DOC]

Additional file1

**Data analysis after exclusion of all patients who underwent more than one EBL procedures from re-entry into data analysis after first ligation session**

Table 6: Clinical characteristics of patients undergoing endoscopic band ligation (n=255).

|  | Without bleeding complications (n = 225) | Bleeding from recurrent varices (n = 15) | Bleeding from EBL-ulcers (n = 14) | Bleeding from an unknown location (n = 1) |
| --- | --- | --- | --- | --- |
| Age (y): | 57.3 ± 12.6 | 53.5 ± 12.2 | 51.1 ± 10.4 | 36 ± 0.0 |
| Sex (M/F): | 146/ 79 | 9/ 6 | 10/ 4 | 0/ 1 |
| Variceal strands: | 3.00 ± 0.8 | 3.1 ± 0.9 | 3.3 ± 0.7 | 4.0 ± 0.0 |
| Average number of applied rubber bands per patient and session: | 6.0 ± 2.4 | 5.7 ± 3.7 | 7.9 ± 2.7 | 9.0 ± 0. 0 |
| Duration of hospital stay after ligation (days) | 13.5 ± 9.0 | 18.9 ± 14.1 | 21.8 ± 15.4 | 18 ± 0.0 |

Table 7: Mode of ligation and source of bleeding in performed ligation sessions.

|  | Elective ligation  (n 128) | Ligation for acute bleeding control  (n = 127) | p |
| --- | --- | --- | --- |
| Bleedings | 8 (6.3 %) | 22 (17.3 %) | 0.007 |
| Bleeding at ligation site | 1 (0.8 %) | 13 (10.2 %) | 0.001 |
| Bleeding at recurrent varices | 7 (5.5 %) | 8 (6.3 %) | NS |
| Esophageal bleeding of unknown location | 0 | 1 (0.8 %) | NS |

Table 8: Time intervals and applied ligation bands.

|  | Elective ligation | Ligations for acute bleeding control |
| --- | --- | --- |
| Duration of hospital stay after ligation (days) | 12,7 ± 8,4 | 15.5 ± 10.9 |
| Time interval between ligation and next endoscopy (days) | 7.6 ± 3.5 | 6.1 ± 3.4 |
| Time interval to detected band drop-off (days) | 9.3 ± 5.1 | 8.5 ± 3.5 |
| Avarage number of applied rubber bands per patient and sessions | 6.1 ± 2.5 | 6.0 ± 2.6 |

Table 9: Number of applied ligation bands per session.

|  | Average of applied ligation bands | p |
| --- | --- | --- |
| Sessions without complications (n = 357): | 6.0 ± 2.4 |  |
| Bleeding complications altogether (n = 30): | 6.8 ± 3.1 | NS |
| Bleeding at ligation site (n = 14): | 7.9 ± 2.7 | 0.006 |
| Bleeding at recurrent varices (n = 15): | 5.7 ± 3.7 | NS |
| Esophageal bleeding of unknown location: | 9 ± 0 | NS |
