# Supplementary figures and images for: Differences in bleeding behavior after endoscopic band ligation: a retrospective analysis
Source: BMC Gastroenterol. 2010 Jan 15;10:5. doi: 10.1186/1471-230X-10-5 (PMC2827370; doi:10.1186/1471-230X-10-5)

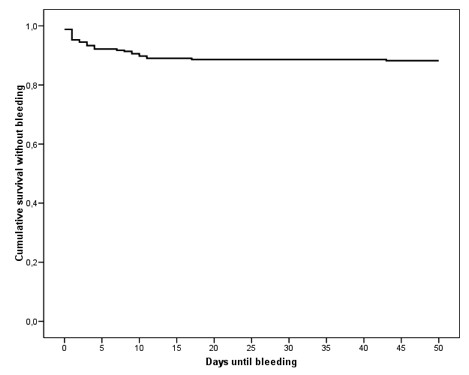

Supplement: Additional file 2 — Kaplan-Meier estimation of cumulative survival without hemorrhage regarding only the first EBL procedure per patient. All patients who underwent more than one EBL procedures were excluded from re-entry into data analysis after first ligation session and Kaplan-Meier estimates were evaluated regarding only the first EBL procedure of each patient. The overall risk of rebleeding after EBL is reduced from 11.8% to 3.9% after four days, and to 0.8% after 11 days. [file 1471-230X-10-5-S2.JPEG]

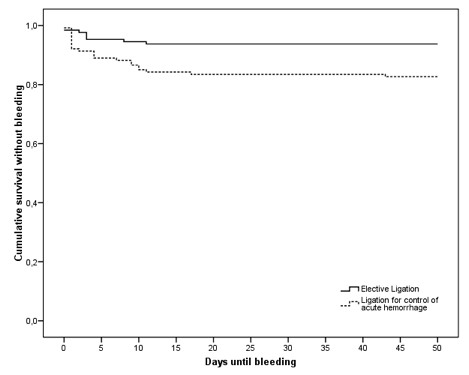

Supplement: Additional file 3 — Kaplan-Meier estimation of cumulative survival without hemorrhage for elective and emergency EBL regarding only the first EBL procedure per patient. All patients who underwent more than one EBL procedures were excluded from re-entry into data analysis after first ligation session and Kaplan-Meier estimates were evaluated regarding only the first EBL procedure of each patient. The bleeding risk four days after emergency EBL is significantly higher than after elective treatment, p = 0.042. [file 1471-230X-10-5-S3.JPEG]
